# Integrating Peak Colocalization and Motif Enrichment Analysis for the Discovery of Genome-Wide Regulatory Modules and Transcription Factor Recruitment Rules

**DOI:** 10.3389/fgene.2020.00072

**Published:** 2020-02-21

**Authors:** Mirko Ronzio, Federico Zambelli, Diletta Dolfini, Roberto Mantovani, Giulio Pavesi

**Affiliations:** Dipartimento di Bioscienze, Università di Milano, Milan, Italy

**Keywords:** ChIP-seq, colocalization analysis, transcription factor (TF), transcriptional regulation, transcription factor binding sites (TFBS)

## Abstract

Chromatin immunoprecipitation followed by next-generation sequencing (ChIP-Seq) has opened new avenues of research in the genome-wide characterization of regulatory DNA-protein interactions at the genetic and epigenetic level. As a consequence, it has become the *de facto* standard for studies on the regulation of transcription, and literally thousands of data sets for transcription factors and cofactors in different conditions and species are now available to the scientific community. However, while pipelines and best practices have been established for the analysis of a single experiment, there is still no consensus on the best way to perform an integrated analysis of multiple datasets in the same condition, in order to identify the most relevant and widespread regulatory modules composed by different transcription factors and cofactors. We present here a computational pipeline for this task, that integrates peak summit colocalization, a novel statistical framework for the evaluation of its significance, and motif enrichment analysis. We show examples of its application to ENCODE data, that led to the identification of relevant regulatory modules composed of different factors, as well as the organization on DNA of the binding motifs responsible for their recruitment.

## Introduction

Next-generation sequencing based assays have opened novel avenues of investigation in every aspect of research in genomics. In particular, they have become the standard in the genome-wide characterization of all the elements concurring to the regulation of gene transcription, like nucleosome positioning (Buenrostro et al., 2013; Pajoro et al., 2018), DNA accessibility (Giresi et al., 2007), DNA methylation, histone modifications and chromatin states (Roadmap Epigenomics Consortium et al., 2015), transcription factor binding (Johnson et al., 2007), and long-distance enhancer-promoter interactions (Fullwood and Ruan, 2009; Lieberman-Aiden et al., 2009).

As a consequence, literally thousands of experiments have been published on one or more of the above aspects in different species and conditions, and large scale projects like ENCODE (Gerstein et al., 2012) and Roadmap Epigenomics (Roadmap Epigenomics Consortium et al., 2015) have been launched. It is now standard practice also for small or midsize labs to produce several datasets with different experiments, and to merge the results obtained into a single overall picture of the regulatory landscape of the condition studied.

Genome-wide NGS-based assays usually produce as a main result a list of genomic regions, where base pairs included in these regions satisfy the condition being tested, e.g., they are nucleosome-free, bound by a transcription factor, occupied by a nucleosome carrying a given histone modification, and so on. Further information can be associated with each region, as for example, its enrichment in the sequenced sample, that can be expressed according to different measures. Key factors for the reliability of the results produced are both wet lab protocols and the downstream bioinformatic analysis of the data, and indeed, as of today, a consensus has been reached for which are to be considered the best practices for both (Landt et al., 2012). However, we are far from having de facto standards for the integrative analysis of the results of different experiments. In principle, a single base pair on the genome appearing in the output of two or more experiments can be considered to satisfy simultaneously the different conditions that have been tested. How this information can be interpreted depends on the experiments producing the data to be analyzed. For example, ChIP-Seq assays for different histone modifications in the same condition can be processed with approaches like segmentation (Ernst and Kellis, 2017), in order to identify their most relevant combinations on the genome, and to produce a genome-wide map of chromatin states each characterized by a different combination of modifications, mapping the location of active or repressed promoters, enhancers, transcribed regions, and so on. However, the integrative analysis of different ChIP-Seq experiments for DNA binding proteins like transcription factors is usually performed with different criteria and principles, often designed *ad hoc* for the protein or condition studied.

In this work we present a simple pipeline for the integrative analysis of any number of ChIP-Seq experiments for transcription factors (TFs) or cofactors. Each ChIP-Seq experiment returns a genome-wide map of the binding locations on DNA of the protein studied. While this phenomenon is usually represented as a single protein interacting with DNA, in reality different factors and cofactors form large protein complexes, binding DNA at distal and proximal regions, that recruit RNA polymerase and initiate transcription. Thus, it is of the utmost importance for obtaining a complete understanding of the mechanisms behind the regulation of transcription not to treat each factor as a separate entity, but to identify combinations of different factors binding DNA as a complex at the same loci of the genome, and evaluate if these coassociations can constitute widespread regulatory modules.

Given the results of ChIP-Seq experiments for any number of different TFs or cofactors, our pipeline has been designed to answer the following questions: (1) Which are the combinations of factors and cofactors that are found with higher frequency on the genome? (2) Are the combinations found actually significant, that is, not resulting from random associations between different proteins but indeed found with high frequency on the genome, thus signaling a higher level of organization in transcriptional regulation? (3) Which are the recruiting rules on DNA, that is, which are the factors actually bound to DNA, and are there specific combinations (e.g., distance or orientation requirements) for their DNA-binding sites?

These questions have become more and more relevant over the years, once large datasets, like the assays performed by the ENCODE project, have been released. Indeed, curated databases containing thousands of ChIP-Seq datasets for TFs in different species and conditions are now publicly available, like ChIP-Atlas (Oki et al., 2018) or ReMap (Chèneby et al., 2018). An important feature of these repositories is that, like in the ENCODE project, all datasets included have been uniformly reprocessed, in order to make their comparison as less biased as possible by different choices in data analysis.

TF colocalization on the genome has been already defined and tacked with different approaches, ever since the introduction of the first NGS-based assays, as for example in (Chen et al., 2008). Several works have addressed the problem by starting from the position of TF binding peaks on the genome [see among many others (Chen et al., 2008; Gerstein et al., 2012)]. Colocalization, and its significance, is then assessed starting from the number of overlapping peaks, and evaluated with explorative or correlation measures like Pearson correlation (Chen et al., 2008), z-scores (Gerstein et al., 2012), the Jaccard index (Salvatore et al., 2019), or with machine learning based techniques like self-organizing maps (Xie et al., 2013).

An orthogonal approach is to analyze regions resulting from a single ChIP-Seq experiment for enrichment of sequence motifs known to represent sites be bound by other TFs, as for example in (Wang et al., 2012; Levitsky et al., 2019). Candidate TFs thus identified can be likely members of the same regulatory module. The pipeline we introduce here is indeed a combination of these two approaches, peak colocalization and motif enrichment analysis, with the additional introduction of a statistical framework designed *ad hoc* to assess both.

## Methods

### Transcription Factor Colocalization

#### Defining Overlapping Peaks and Cobinding Regions

The overlap among two or more genome-wide datasets can formalized in different ways, both in the definition of common features and in the evaluation of the significance of the overlap found, as reviewed for example in (Kanduri et al., 2019; Salvatore et al., 2019).

**Figure 1:**
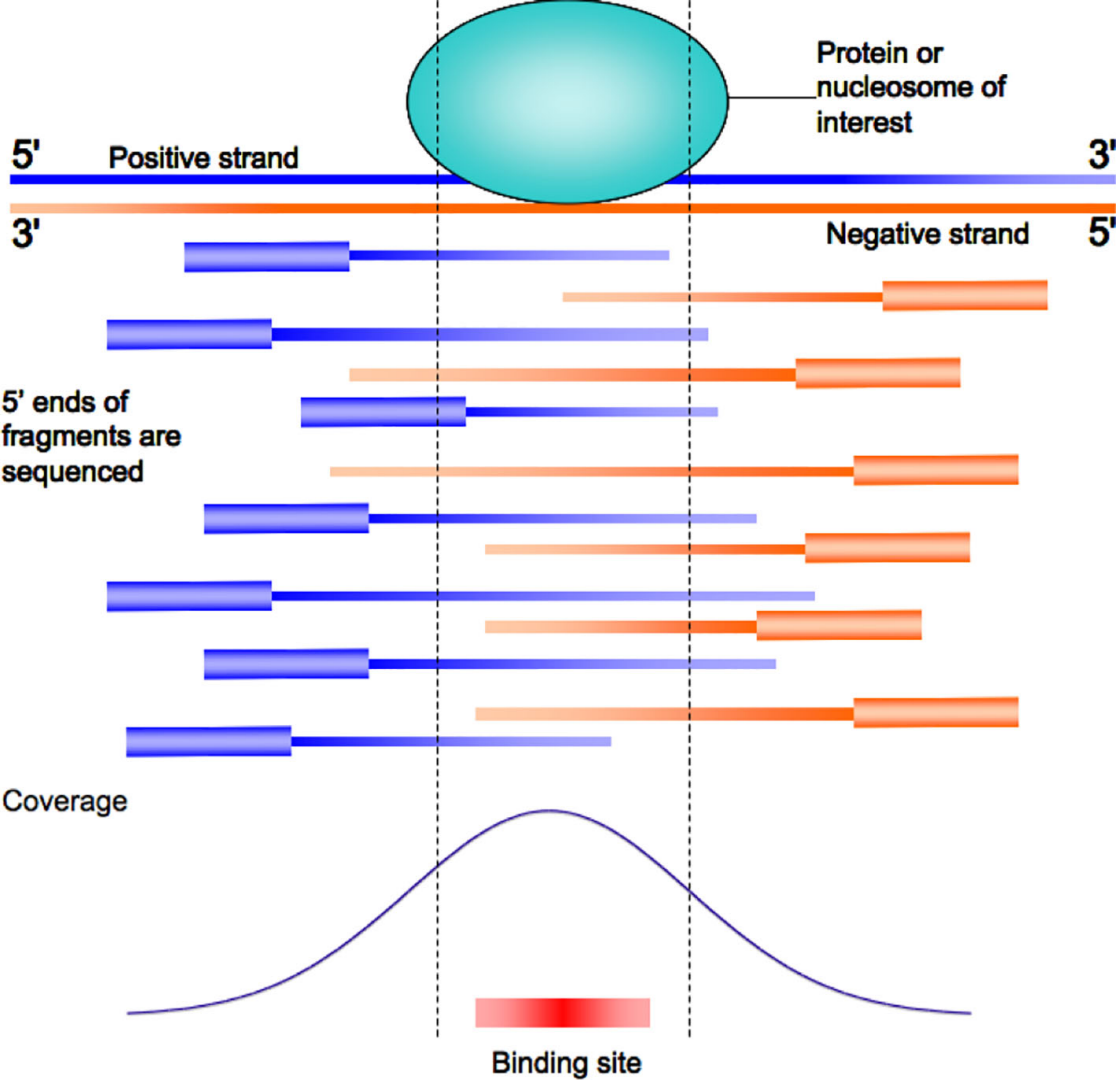
The typical peak shaped enrichment plot for a ChIP-Seq experiment resulting from read mapping on the genome. The actual point of contact of the protein studied on DNA is usually close to the point of maximum local enrichment (peak summit).

ChIP-Seq experiments for DNA binding proteins like TFs produce as output enriched regions usually called “peaks.” This is due to the experimental protocol preparing the DNA to be sequenced (**Figure 1**). The fragments, produced by random DNA sonication, are usually of about 200 bps. Once mapping on the genome of the sequenced reads has been performed, the actual fragment size can be reestimated according to the distance between clusters of reads mapping on opposite strands, (Zhang et al., 2008; Bailey and MacHanick, 2012; Mathelier et al., 2016) in order to improve the resolution obtained by the experiment. The result is a coverage “signal” map, giving an estimate of how many times each base pair of the genome was present in the sequenced DNA sample. Since the actual point of interaction between the protein studied and DNA is present in each of the fragments, enriched regions show a typical “peak” shape in the signal map (**Figure 1**).

Algorithms for “peak calling” thus return regions where the observed enrichment and respective signal is not considered to be due to random experimental noise (Thomas et al., 2017). A typical region is reported to be a few hundreds of base pairs long, while the sites actually bound by TFs are much smaller, usually no more than 10–12 base pairs. However, the local maxima of the peaks correspond, or at least are not too distant from, the actual binding site of the protein studied (Zhang et al., 2008). Indeed, ChIP-Seq peaks usually show a good “centrality,” that is, the likely binding site of DNA returned by sequence analysis is usually found to be within a few dozen base pairs from the summit (Zhang et al., 2008; Bailey and MacHanick, 2012; Mathelier et al., 2016). For proteins like cofactors, not directly in contact with DNA, the argument still holds, with the only difference that summits and binding sites are related to the protein(s) of the complex tethering the cofactor on DNA.

The above considerations should be kept in mind when performing colocalization analyses for TF binding. Simply defining a DNA locus as “cobound” by two different transcription factors if two peak regions overlap might correspond to cases in which the actual binding sites of the factors are hundreds of base pairs apart. Thus, our approach to defining two (or more) TFs or cofactors as “colocalizing” on the genome is based instead on peak summits coordinates. In other words, we do not require two peak regions just to overlap, but we consider the location of the respective summits. We then define two peaks as “overlapping,” and the TFs to bind DNA in close proximity, if the respective summits are within *d_s_* base pairs from one another, with an approach similar to (Chen et al., 2008), where the “center” of peak regions was employed to assess colocalization. With respect to (Chen et al., 2008), however, we introduce also a statistical assessment of the significance of overlaps, as detailed in the next section. As a default threshold for this step we set as maximum distance *d_s_* = 150 bps, a distance commonly employed in studies of this kind (Wang et al., 2012), which also makes the calculation of the statistical significance of overlap straightforward as shown in the next section.

**Figure 2:**
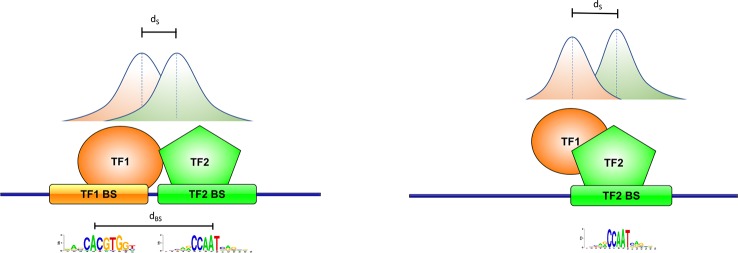
Definition of cobinding peaks. Peak summits are usually close of the point of contact of the corresponding transcription factor (TF) with DNA. Two peak summits within a given number of base pairs *d_s_* (150 in this work) should thus correspond to two TFs binding DNA in close proximity with one another (left, with the respective binding sites within *d_bs_* base pairs), or to one of the two TF tethering the other on DNA (right).

We define pairs of peaks satisfying this criterion as *cobinding* peaks. This, in turn, usually corresponds to having the binding sites on DNA of the two factors located within a number of base pairs (*d_bs_*) not too much different from the *d_s_* distance. Or, alternatively, summit proximity could be due to only one of the two factors in contact with DNA, with the other one being anyway part of the same complex (**Figure 2**).

#### Assessing Statistical Significance of Peak Overlaps

Let TF1 and TF2 be two TFs on which the cobinding analysis is performed; let *n* and *m* the respective number of peaks, and *k* the number of cobinding peaks defined as at the previous point. We want to assess the probability of finding by chance *k* cobinding peaks (hence, regions bound by both TFs), given *n* and *m*.

In our approach, we also define a constant *N*, denoting the number of regions across the genome available for TF binding, whose size equals our “cobinding” region size of 150 bps. A straightforward way to estimate *N* would be to count the overall number of regions bound by all the TFs active in the condition studied. This approach, however, would have the effect of underestimating *N* if binding data were available only for a limited number of TFs, i.e. several regions would not be included in the count simply because the ChIP-Seq experiments for the TFs binding them had not been performed.

However, since a region bound by a TF usually corresponds to accessible DNA, estimate for *N* can be obtained from the number of nucleosome-free regions, and their respective size. Thus, we took advantage of maps of accessible DNA produced in several different cell lines in the framework of the ENCODE project, through digital genomic footprinting (Sabo et al., 2004; Vierstra and Stamatoyannopoulos, 2016). The advantage of these datasets (retrieved from the UCSC genome browser track “UW DNaseI DGF” on the GRCh37 assembly) is that the genome is split into regions of exactly 150 bps, that corresponds to the maximum *d_s_* distance between summits we allow for peak overlap. Thus, cobinding peaks can be seen as two peak summits falling exactly within the same accessible region. The value of *N* is naturally cell- and condition-specific, ranging roughly from 200,000 to 250,000 for most of the ENCODE cell lines on which this assay has been performed. In case this number is available for the condition studied it can be thus employed in a straightforward way. If not, we advise to employ N = 250,000, a value we consider to be reliable enough for all different conditions in human, and also in other mammalian genomes like mouse. The only exception to this rule are embryonic stem cell lines, in which nucleosome occupancy has been shown to be significantly lower (Celona et al., 2011; Harwood et al., 2019), with an average number of genomic loci available for binding almost doubled. Thus, for these the suggested value is N = 500,000.

Indeed, the vast majority of the accessible regions results to be bound by TFs (80%–90% in the different ENCODE cell lines for which digital footprinting data are available). Once again, the sole exception are stem cell lines, where the percentage is lower (70% in ENCODE H7-ESC cells), also because less TF ChIP-Seq experiments are available for this condition. The number of regions actually bound (more than 300,000) is anyway larger, nearly twice as much as the other cell lines.

Similar estimates can be derived for other species and taxa, since nucleosome occupancy and DNA accessibility data are available for all the most widely studied species, as for example in Drosophila (Thomas et al., 2011), or Arabidopsis thaliana (Tannenbaum et al., 2018). In these two species the smaller genome size (hundreds of millions of base pairs) in turn results in a proportionally lower number of estimated accessible regions (tens of thousands).

An alternative, if data are available, could be to focus on regions annotated as active promoters or enhancers, as revealed by presence of specific histone marks or resulting from a genome segmentation approach like ChromHMM (Ernst and Kellis, 2017). An estimate for *N* can be thus derived by the number of regions of size *d_s_* found annotated as active promoter or enhancer. Also, the overlap between two TFs can be assessed in either subset of regions, thus identifying promoter- or enhancer-specific modules.

Once an estimate for *N* has been produced, the probability of finding *k* cobinding peaks for two TFs by chance given *n* and *m* (the number of peaks for the two TFs, respectively) can be computed with different approaches, for example with the hypergeometric distribution:

$$p\left(k;n,m,N\right)=\frac{\left(\begin{array}{c} n\\ k \end{array}\right)\left(\begin{array}{c} N-n\\ m-k \end{array}\right)}{\left(\begin{array}{c} N\\ m \end{array}\right)}$$

or the Poisson distribution:

$$p\left(k;n,m,N\right)=\frac{e^{-\lambda }\lambda^{k}}{k!}$$

where $\lambda =\frac{mn}{N}$. In our experiments we employed the latter, since the p-values returned are more conservative. Since both distributions are two-tailed, low p-values can point to significant colocalization across the genome (*k* higher than the expected value), or vice versa if *k* is lower than the expected value than the two TFs considered tend to avoid one another on the genome.

This analysis is performed on every pair of experiments available. If several pairwise comparisons are performed, then the p-values should also be corrected for multiple testing. For example, in the results we present here we analyzed 329 ENCODE datasets for TFs in the K562 cell line, thus with 329 × 329 pairwise comparisons. We employed once again the most conservative choice, the Bonferroni correction, multiplying the p-values by 329^2^ = 108,241.

#### Building Modules With More Than Two Factors

The results of the pairwise comparisons described at the previous step can be further extended to modules composed by more than two TFs or cofactors.

An initial explorative analysis (see Results) is to define a colocalization score for each pair of experiments *i* and *j*, starting from the corresponding number of cobinding peaks *k*, and the respective p-value *p*_ij_, as -log_10_ *p*_ij_ if the observed overlap is higher than the expected value, log_10_ *p*_ij_ (and hence a negative number) otherwise. The resulting values can be employed to represent the results as a heatmap, and clustering the heatmap can in turn highlight groups of TFs with significant pairwise overlaps, hence likely to be found together in the same regulatory module.

Another approach we introduce is to choose a “base” TF_b_, and determine whether other TFs tend to colocalize within its peaks. For every pair of TFs (TF_i_ and TF_j_) different from TF_b_, this step is formalized as follows:

- Let *k_b_* the number of peaks for the base TF_b_;
- Let *k_i_* and *k_j_* the number of cobinding regions with TF_b_ of the two other TFs (TF_i_ and TF_j_);
- Let *k_ij_* be the number of cobinding regions for both TF_i_ and TF_j_ with TF_b_

At this point, the significance of the cobinding of TF_i_ and TF_j_ in correspondence to TF_b_ binding sites can be assessed again with a statistical test as in normal pairwise comparison. That is, we can compute the probability of finding *k_ij_* cobinding regions for TF_i_, TF_j_ and TF_b_, given *k*_i_, *k*_j_, and *k_b_*. The p-value can be computed again with a Poisson distribution:

$$p\left(k_{ij};k_{i},k_{j},k_{b}\right)=\;\frac{e^{-\lambda }\lambda^{k_{ij}}}{k_{ij}!}$$

where $\lambda =\frac{k_{i}k_{j}}{k_{b}}$.

The resulting p-values can in turn be converted again into scores, with the respective clustering highlighting groups of TFs colocalizing, but this time with TF_b_ as “tether” on DNA. Once the base TF_b_ has been chosen, this step can be performed by selecting only TFs that had a significant overlap with TF_b_ at the previous step in their pairwise comparison with it.

If necessary, this step can be iterated any number of times, e.g. assessing the significance of the overlap of a fourth TF given the cobinding regions of TF_b_, TF_i_, TF_j_, and so on.

#### Defining Binding and Recruitment Rules Through Motif Analysis

Once a list of genomic regions bound simultaneously by two (or more) TFs has been produced, the next step is to determine if the respective binding sites are actually present on DNA, and if so if they present any arrangement, e.g., are found at a precise distance, further hinting at co-operative binding and interactions between the respective proteins.

The binding specificity of a TF is usually defined with a *position specific frequency matrix*, or *profile*, obtained by the alignment of a collection of binding sites for the TF (Stormo, 2000; Zambelli et al., 2013a), defining its nucleotide preference on DNA. Several collections of profiles are freely available, derived from large-scale *in vitro* assays like SELEX or by the application of motif discovery tools to ChIP-Seq peak regions (Wingender, 2008; Mathelier et al., 2016; Khan et al., 2018; Wingender et al., 2018). For example, the latest version of the JASPAR database (Khan et al., 2018) includes for human and mouse profiles derived from the analysis of the ENCODE datasets.

This step can be formalized as a *motif enrichment analysis*, that is, the regions are analyzed in order to determine whether the motif representing the binding specificity of each of the TFs involved can be considered to be enriched in them, both in number and quality of instances found. Different tools have been introduced for this task, including a tool we developed called PscanChIP (Zambelli et al., 2013b). Since, as we previously discussed, the region more likely to correspond to the actual point of contact of the TF on DNA is located near peak summits, PscanChIP requires as input a list of one base pair peak summit coordinates, and scans the region of 150 base pairs centered on each one employing a collection of motifs like the JASPAR database or defined by the user.

Actual motif enrichment is evaluated by the tool in different ways:

- Global enrichment: enrichment is assessed with respect to a genomic background, that is, motifs are overrepresented in the selected regions with respect to the rest of the genome accessible to TF binding. Hence, a motif with significant global enrichment could correspond to the actual binding site of the TF (usually the most significant one), or binding sites of other TFs which show a clear genome-wide tendency to bind in association with it.
- Local enrichment: the enrichment of the motif in the peak summit regions is compared to the regions immediately upstream and downstream of the summit regions themselves;
- Positional bias: the localization of the most likely instance of the motif in each summit regions is identified, and the resulting distribution is compared to a theoretical uniform distribution.

Thus, the results of motif enrichment analysis can be interpreted as follows: if a motif corresponding to one of the TFs binding the regions selected is found to be significantly enriched according to the global p-value reported by PscanChIP, then the corresponding TF can be assumed to be in contact with DNA. Since the regions submitted as input are bound *in vivo* by one or more TFs, then the corresponding motifs should be the ones with the lowest global p-values among those employed in the analysis. Also, given the centrality of ChIP-Seq peaks, they should present a positional bias towards the middle of the regions. Otherwise, TFs for which no significant motif enrichment is found can be considered not to be directly binding DNA, although part of a complex in contact with DNA (**Figure 2**).

There are a few main differences between PscanChIP and other methods for the same task. The presence/absence of a motif instance in a region is evaluated with a score, ranging from 0 to 1, instead of a yes/no decision (binding motif present/absent) as for example in recent works (Dergilev et al., 2017; Czipa et al., 2020; Levitsky et al., 2019), which are also focused on the analysis of regions surrounding ChIP-Seq summits. Mean and variance of scores of best motif instances in each of the summit regions are in turn employed by PscanChIP to assess motif enrichment not only with respect to regions flanking peaks (local enrichment), as in similar tools (Zhang et al., 2011; Bailey and MacHanick, 2012), but also with respect to the rest of the genome, providing a more accurate evaluation of their significance.

PscanChIP also permits to perform a “motif centered” analysis. Once the first round of motif enrichment analysis has been completed in the neighborhood of peak summits, users can select one of the motifs resulting to be significantly enriched, and rerun the analysis centered this time on the most likely instance of the motif in each of the input regions. Regions containing a low quality instance for the motif chosen are automatically discarded. The idea is to replace the peak summit for the TF of interest with the most likely location of its actual binding site on DNA. Thus, if two or more TFs have their respective binding sites enriched in the regions, then the motif centered analysis is meant to highlight if there is also a preferential arrangement of their sites in the regions, signaled by the “positional bias” p-value output by PscanChIP. This fact is in turn a strong indicator that the corresponding TFs are likely to interact, require a precise arrangement for their binding sites on DNA, and thus influence the respective recruitment on DNA. Thus, by submitting to PscanChIP cobinding peak regions for two of more TFs, we can assess the enrichment and relative positions of the respective binding sites.

Given a set of ChIP-Seq experiments for TFs and cofactors, and the corresponding peaks and summits, our pipeline can be thus summarized in the following steps:

1. Compute the summit neighborhood overlap for each pair of TFs, and the corresponding p-values;
2. convert the p-values into scores, and cluster the experiments according to the scores; this step is optional, but provides a quick overview of the results obtained;
3. for selected pairs of TFs, define the recruitment and binding rules on DNA by submitting the list of peak summits of either one falling in cobinding regions to PscanChIP:
  - If motifs for both TFs are found to be significantly enriched according to the global p-value, assess whether there is a preferential arrangement or spacing of the corresponding binding sites through motif centered analysis on either one, by checking whether PscanChIP reports a significant positional bias p-value (< 0.01) for the other; if so, the distribution of the distances between the two motifs can be further analyzed, starting from the relative motif position in each of the input regions reported by PscanChIP.
  - If only one motif is found to be enriched, then the corresponding TF can be considered to be recruiting the other on DNA.
  - If neither motif is enriched, then either the motifs employed are not correct for the TFs studied, or there might exist a third factor responsible for the recruitment of the two factors considered.
4. The previous steps can be iterated in order to find significant triplets, quadruplets, and so on, of TFs, and the corresponding binding sites on DNA.

Peak cobinding analysis can be easily implemented with in-house scripts, or with utilities like bedtools (Quinlan and Hall, 2010). A shell script making use of the bedtools “intersect” function (bedtools version 2.29) is provided as **Supplementary File 1**.

PscanChIP is available both through a dedicated web interface, or as a standalone software package. Both already contain the latest release of the JASPAR database. Users can anyway add to the already present collection their own profiles, e.g., the result of a motif discovery analysis on the regions of ChIP-Seq experiments with tools like MEME (Machanick and Bailey, 2011), HOMER (Heinz et al., 2010) or Weeder (Zambelli et al., 2014). Histograms of motif distance distributions presented here were produced by plotting the relative distance between two motifs as output by the motif centered analysis on one of the two of PscanChIP.

## Materials

ChIP-Seq data (“optimal thresholded” peak and summit coordinates) for 492 experiments of transcription factors of cofactors in the K562 cell line were retrieved from the ENCODE data repository (Davis et al., 2018) (www.encodeproject.org) as of 31^st^ December of 2018. Each experiment has been performed in at least two replicates, whose consistency has been checked according to different metrics. Only experiments with consistent replicates have been released by ENCODE, with replicates merged into a single list of consensus peak and summit coordinates (Landt et al., 2012).

Since in some cases for the same TF data contained more than one experiment (e.g., with different tagging or antibodies, with or without stimulation of the cells), we filtered the datasets as follows: (1) Experiments on stimulated cells were not considered. (2) In case for the same TF experiments were performed with antibodies against both the wild-type protein and a tagged protein (e.g., with flag or GFP), only the former was kept. Finally, in case of redundant experiments for the same TF not satisfying any of the above conditions we proceeded as follows: (a) if an experiment contained less than 10,000 peaks, and less than half of the peaks of the other(s), it was discarded; (b) if the overlap among the remaining experiments was above 66% we kept the one with the highest number of peaks; otherwise all the experiments for the TF were discarded. Peak overlap was defined as for cobinding peaks, that is, the respective summits had to be located within 150 bps.

After filtering, we obtained non redundant experiments for 329 TFs and other DNA binding proteins. The resulting list, with the respective ENCODE identifiers, is available as **Supplementary Table 1**.

Sequence analysis was performed with PscanChIP version 1.3 (Zambelli et al., 2013b) using the JASPAR 2018 collection of binding sites profiles (Khan et al., 2018), and the K562 background.

## Results

A preliminary version of the pipeline we present had been applied to a comprehensive analysis of ENCODE ChIP-Seq data for transcription factors and cofactors in three different cell lines, focused on modules containing transcription factor NF-Y (Dolfini et al., 2016). NF-Y is a trimeric TF composed of two histone-like subunits (NF-YB and NF-YC) and a sequence-specific subunit (NF-YA) binding to the CCAAT motif (CCAAT box). The main difference of our previous work with the pipeline we present here is that, since the original study was focused on NF-Y, motif enrichment analysis was performed as a preliminary step, and cobinding peaks and binding rules were further investigated only for those TF whose binding regions were enriched for the CCAAT box motif. Here, instead, motif enrichment is assessed as a final step, so to include in the pipeline the analysis of colocalization and recruitment for factors not directly contacting DNA.

We consider NF-Y an excellent case study for several reasons. Its binding sites are functionally very well characterized from the genetic point of view, are in general important, and in some cases their presence in promoters is outright essential for the transcription of the corresponding genes. The binding site motif has a high information content, spanning 5 base pairs flanking the central CCAAT, for a total of 10 discriminatory base pairs. The motif is specific for only one complex, hence avoiding the daunting task of disentangling subtle differences in binding preferences among members of large TFs families.

ENCODE data contain experiments for two of the subunits of the complex (NF-YA and NF-YB) in three cell lines. In each cell line, the number of peaks for NF-YA is always lower than NF-YB, and virtually all peaks for NF-YA overlap a peak for NF-YB. A more detailed analysis revealed that the peaks identified for NF-YB only indeed correspond to “quasi-peaks” for NF-YA, that present enrichment levels below detection thresholds for the bioinformatic tools employed. The conclusion was thus that the NF-YB antibody is more efficient than the one for NF-YA, and that the two subunits can be assumed to be found together bound on DNA, as further discussed in (Fleming et al., 2013). We thus employed peaks for NF-YB for our analysis as representative of the whole NF-Y trimer.

Since the original release, new datasets have considerably expanded the ENCODE repository, for new TFs or new cell lines. Furthermore, while the initial ENCODE release contained datasets processed with different tools and strategies, all ChIP-Seq datasets have been reprocessed with a unique bioinformatic pipeline, applying also more stringent quality controls for experiments to be included in the official release. The result is that some of the TFs originally included in the early ENCODE releases -and in our study- have been removed, or the original peak lists changed, both in number of peaks, peak size, or genomic coordinates of peak regions. We thus reprocessed the new datasets, focusing on the K562 cell line, with our pipeline (see also Materials).

**Figure 3:**
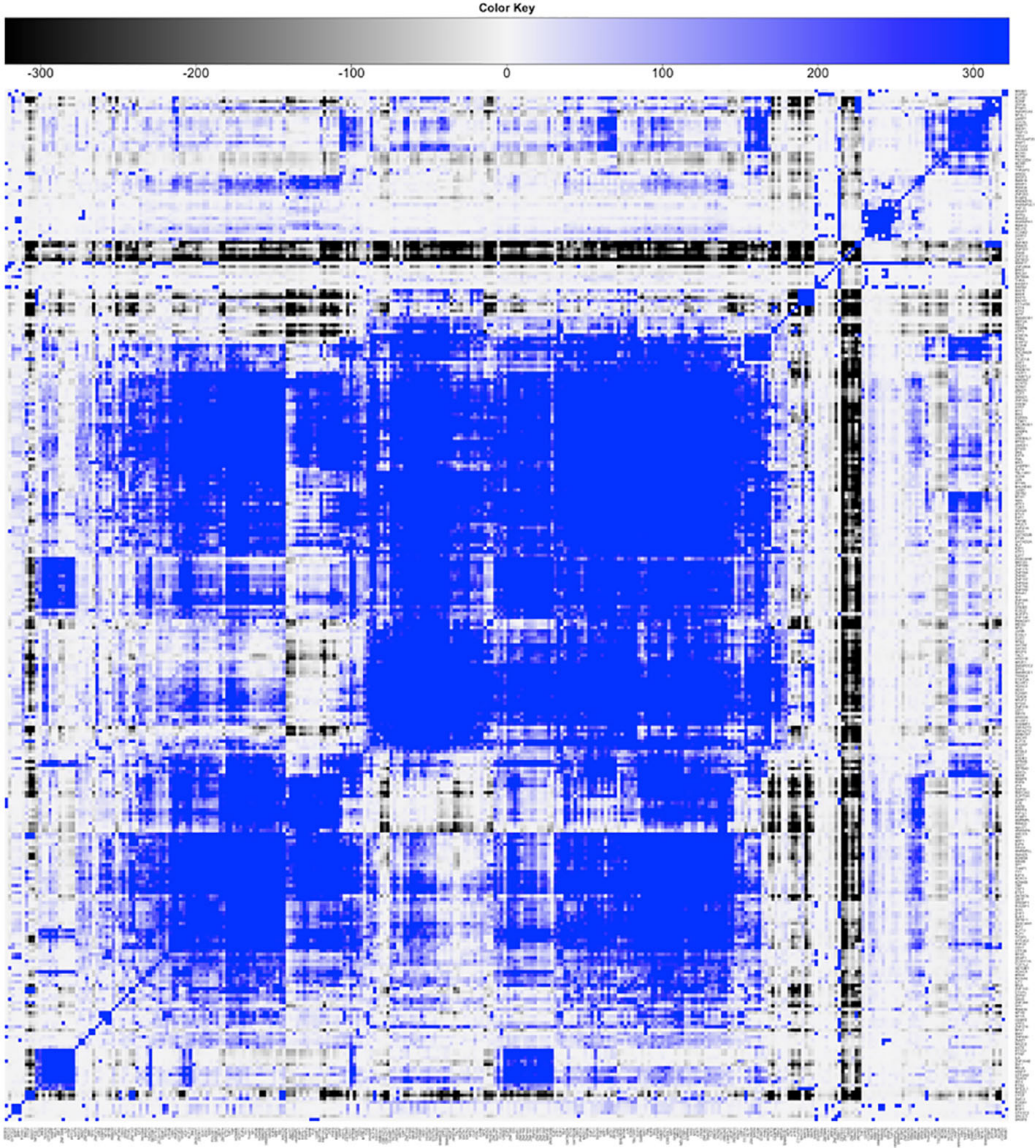
Result of step 1 of the pipeline. Clustered heatmap of pairwise coassociation scores among 329 ENCODE ChIP-Seq experiments in the K562 cell line. Coassociation scores are defined as −log10 of the p-value if the overlap is higher than expected, log10 of the p-value otherwise. Pearson correlation was employed as distance for clustering.

An updated version of the results is summarized in **Figure 3**. The heatmap shows the significance of pairwise cobinding between the ENCODE experiments available for the K562 cell line (result of step 1 of the pipeline). The values represent the log10 of the p-value resulting by the statistical assessment of the overlap significance. Blue colors represent overlap higher than expected (-log10 of the p-value), vice versa for black (log10 of the p-value). Four main large clusters are clearly identifiable in the center of the heatmap, formed by general transcription factors as well as promoter-binding TFs. Several smaller clusters however emerge, composed by proteins binding DNA at distal regions away from genes. The complete results are available as **Supplementary Table 2**.

**Figure 4:**
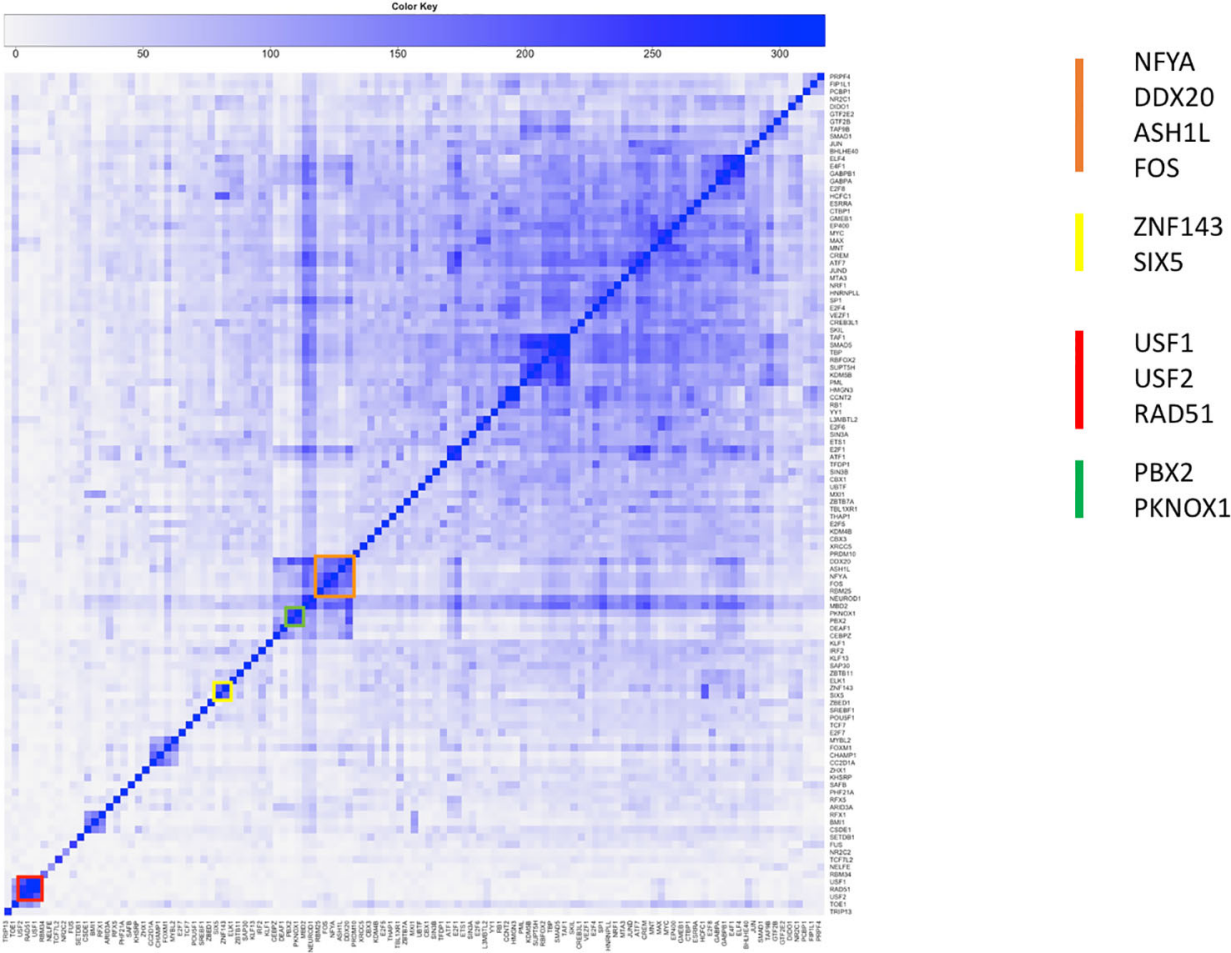
Clustered heatmap of pairwise coassociation scores restricted to peaks cobinding with NF-YB for transcription factors (TFs) with significant overlap with NF-YB (p-value lower than 10^-10^). Pearson correlation was employed as distance for clustering.

**Figure 4** shows the significance of the number of cobinding peaks between pairs of TFs within NF-YB peaks (result of step 2), restricted only to those TFs that had a significant overlap with NF-YB at the first step (enriched with p-value < 10^-10^). It can be observed how several small clusters emerge, clearly identifiable along the main diagonal, each corresponding to a potential genome-wide regulatory module composed by NF-Y and other factors and cofactors.

The already identified module containing NF-Y, FOS, and other factors (Dolfini et al., 2016) was confirmed by the new analysis on the reprocessed data (cluster in orange in **Figure 4**). The presence of NF-YA, which colocalizes with NF-YB as a rule, highlights the significance of this cluster, that is, it covers a significant fraction of the NF-Y binding sites on the genome. FOS is known to form a dimer with JUN, and to bind DNA on the AP1 motif. The surprising result of our analysis was that in the regions of FOS/NF-Y overlap the AP1 motif is not enriched, but indeed seemed to be avoided (under-represented according to PscanChIP), with the CCAAT box bound by NF-Y as the most enriched one. Vice versa, FOS summits not overlapping with NF-Y had the expected AP1 as the most enriched motif. Motif centered analysis on the NF-Y/FOS cobinding peaks identified a second binding motif for NF-Y, with the two CCAAT boxes located with precise spacing on DNA, hinting at two NF-Y molecules forming a complex with FOS (Dolfini et al., 2016; Zambelli and Pavesi, 2017). The same conclusion has been confirmed by independent studies, leading to the interesting hypothesis of a single complex connecting enhancers bound by JUN/FOS to a promoter bound by NF-Y (Haubrock et al., 2016).

**Figure 5:**
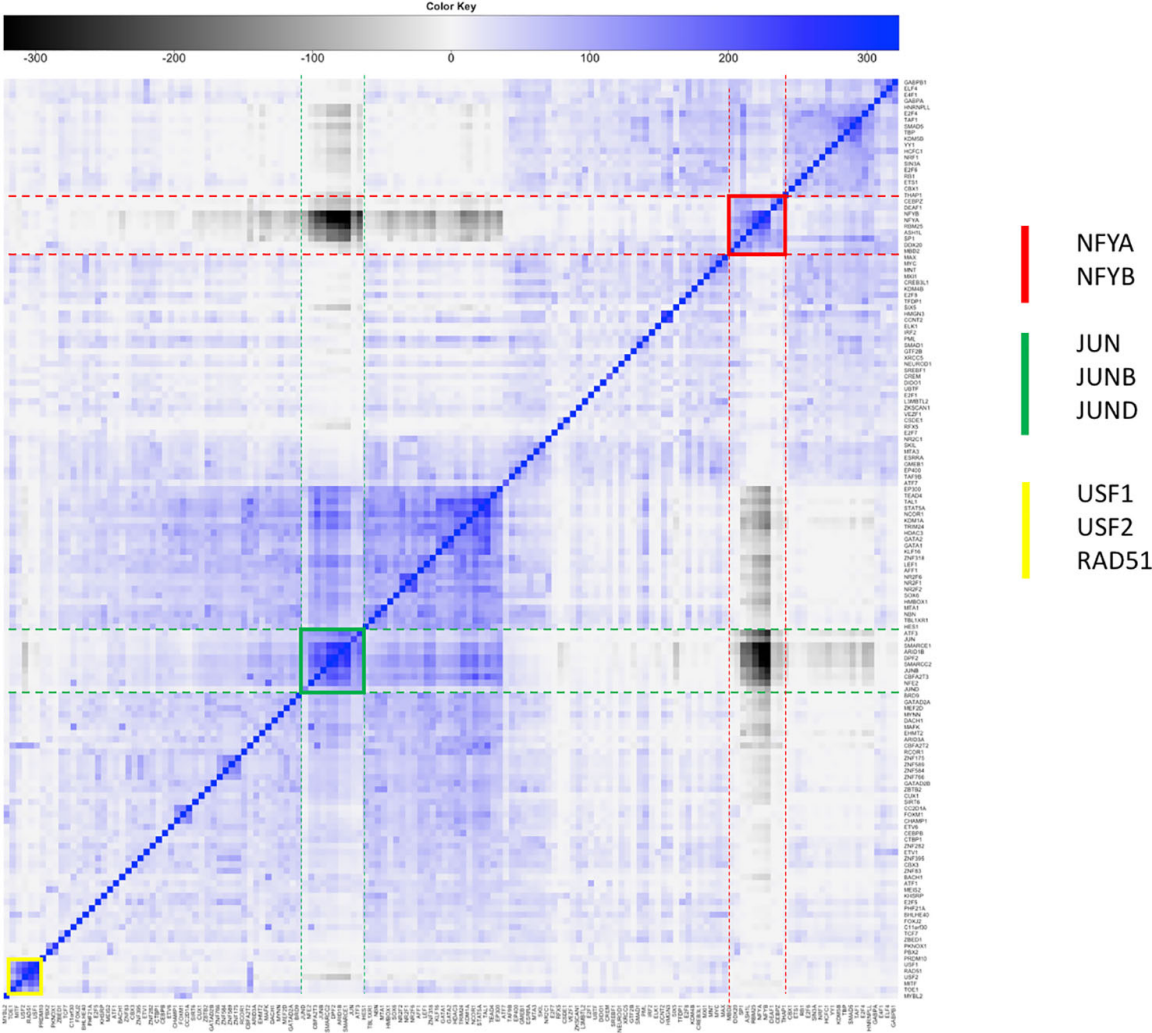
Clustered heatmap of pairwise coassociation scores restricted to peaks cobinding with FOS for transcription factors (TFs) with significant overlap with FOS (p-value lower than 10^-100^). Pearson correlation was employed as distance for clustering.

To further substantiate these results we repeated the analysis of step 2 computing the significance of cobinding peaks between pairs of TFs within FOS peaks, shown in **Figure 5**. Two distinct clusters are easily identifiable, one (highlighted red in the figure) composed by NF-YA/NF-YB and the other factors clustering with NF-Y and FOS in the previous analysis. The second one (green in **Figure 5**) is composed by factors forming the canonical AP1 complex (JUN/JUNB/JUND). The two clusters and clearly separated, and, more interestingly, the members of each show a significant under-representation of their overlap with the others. In other words, the number of cobinding peaks between pairs of members belonging to different clusters is significantly lower than expected, converted into negative scores represented in grayscale in the heatmap. The overall message thus becomes clear: FOS is recruited on DNA by forming a complex either with NF-Y or with JUN factors, but never by both. In fact, when members of either cluster are found with FOS the others are avoided, and vice versa.

**Figure 6:**
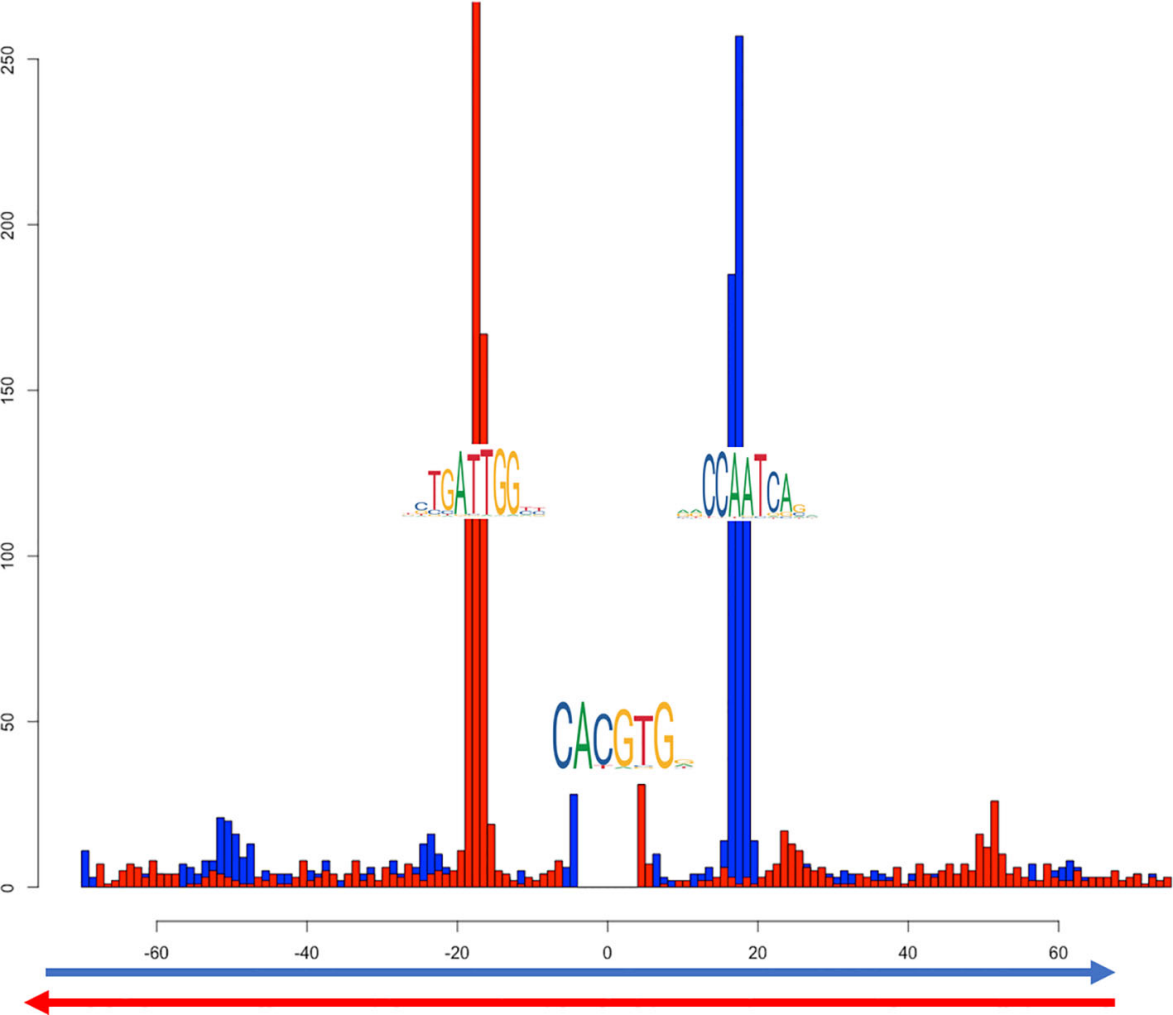
Distribution of distances between the most likely instances of the CCAAT box and the E-box in cobinding peaks of NF-YB with USF1, as reported by PscanChIP from in the motif centered analysis on the USF1 motif (**Supplementary Table 6**). The blue histogram shows the distribution of the position of the CCAAT box when found on the positive strand of the genome; red when on the negative strand. The origin of the x-axis corresponds to the center of the USF1 binding sites (E-box). The analysis has been performed on 2748 cobinding regions for NF-YB and USF1.

Another cluster (in red in **Figure 4**) shows the overlap of NF-Y with both USF1 and USF2, generalizing to the whole genome previous observations (Zhu et al., 2003). USF factors in turn show a significant colocalization within NF-Y peaks together with RAD51. In this case, the motif enrichment analysis for both the NF-Y/USF1 and NF-Y/USF2 cobinding regions returns both the CCAAT-box and the expected E-box as significantly enriched, with a strikingly precise spacing and orientation between the two (shown in **Figure 6** for NF-Y/USF1 cobinding peaks). In most of the cobinding regions, the CCAAT is located downstream of the E-box, at 17 or 18 bps of distance.

**Figure 7:**
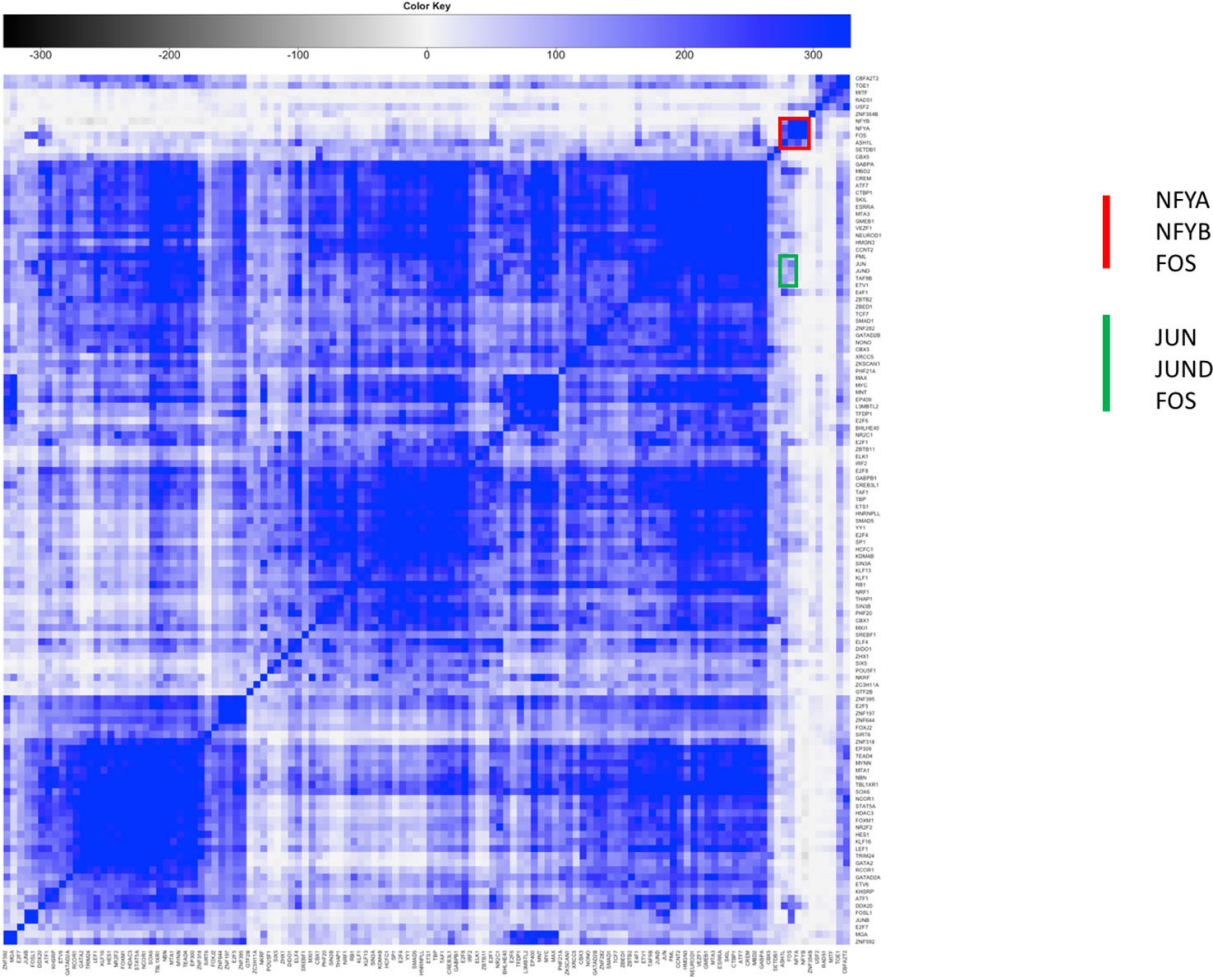
Clustered heatmap of pairwise coassociation scores restricted to peaks cobinding with USF1 for transcription factors (TFs) with significant overlap with USF1 (p-value lower than 10^-100^). Pearson correlation was employed as distance for clustering.

Interestingly, the USF1/USF2 cluster emerges in coassociation with FOS as well (highlighted in yellow in **Figure 5**). Indeed, the interactions of FOS with USF1/2 has been known ever since the discovery of the latter (Blanar and Rutter, 1992; Aperlo et al., 1996). The USF cluster in **Figure 5** does not shows significant overlap with either the NF-Y or the AP1 cluster. Thus, to determine whether FOS colocalizes with USF1/2 with or without NF-Y, we performed another cobinding analysis centered on USF1 peaks (**Figure 7**). Here several clusters emerge, and, strikingly, one small cluster composed exactly bby NF-YA, NF-YB, and FOS (highlighted in red in **Figure 7**). However, the cobinding of FOS with JUN in USF1 peaks is also significant, although JUN clusters elsewhere with members of the AP1 complex (green in **Figure 7**).

By combining the results obtained from the three different points of view just studied, the overall picture emerges. FOS can be recruited either by NF-Y or as a member of the AP1 complex with JUN factors, and the two modes are mutually exclusive. When USF1 is found on DNA together with NF-Y or FOS, it is in general with USF2; when FOS is bound on DNA with USF1/2, it is mainly found in the NF-Y complex, but not exclusively; that is, USF1/2 can be found in a smaller, but significant number of genomic loci also in association with the AP1 complex containing FOS. Complete cobinding statistics for the three TF centered analyses are available as **Supplementary Tables 3**–**5**.

**Figure 8:**
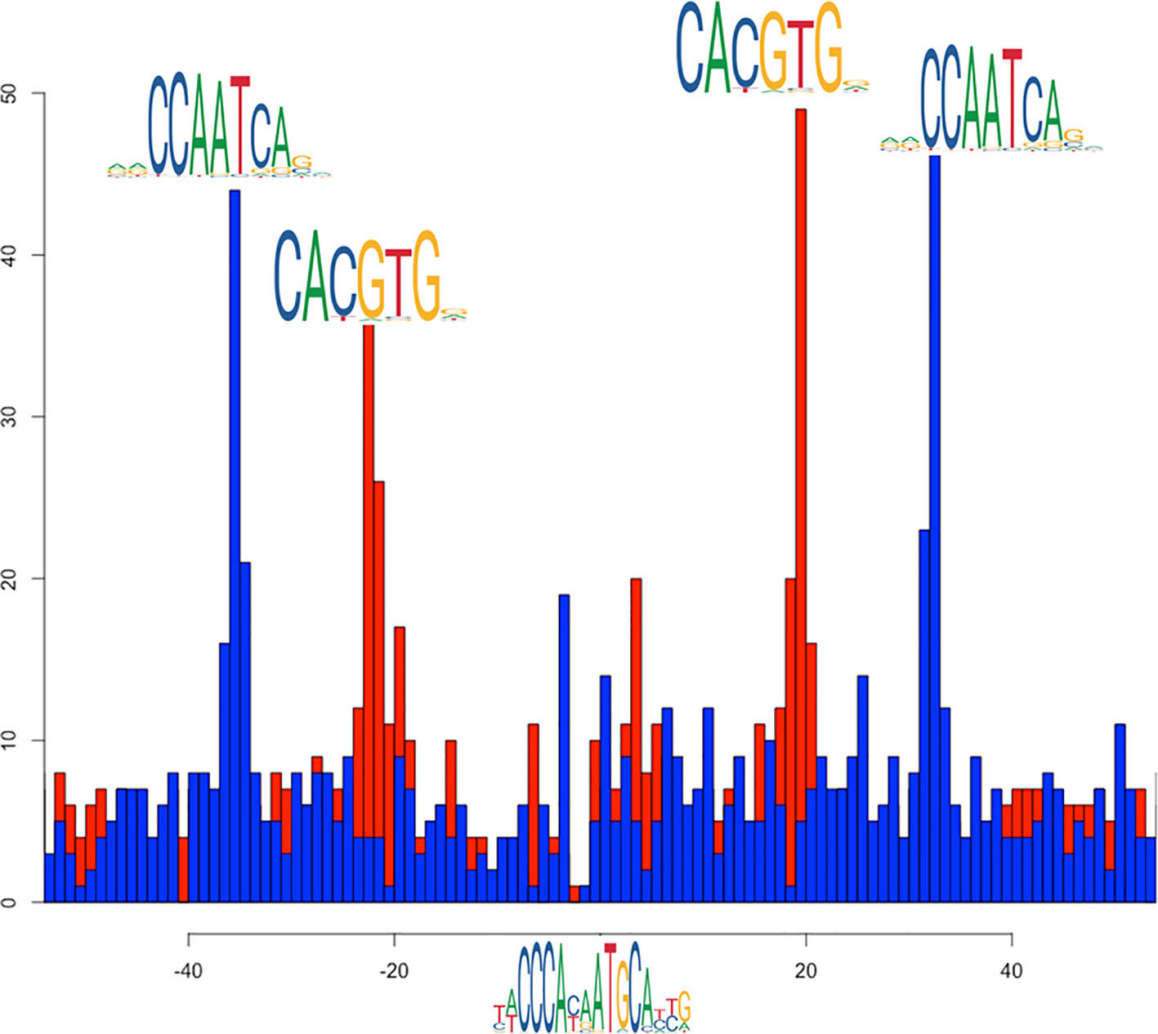
Distribution relative positions of the CCAAT box (blue histogram) and the E-box (red histogram) around the most likely instances of the SIX5/ZNF143 binding site in the cobinding peaks of NF-YB with ZNF143, as reported by PscanChIP from in the motif centered analysis on the ZNF143 motif (**Supplementary Table 7**). The origin of the x-axis corresponds to the center of the ZNF143 binding sites. The analysis has been performed on 1424 cobinding regions for NF-YB and ZNF143.

Another example of combinations of factors colocalizing with NF-Y is the SIX5/ZNF143 pair (yellow cluster in **Figure 4**). The CCAAT box and the ZNF143 binding motifs show evident preferential spacing (**Figure 8**). Sequence analysis also returned significant enrichment and positional bias for an additional E-box motif, also plotted in **Figure 8**, located in between the ZNF143 and CCAAT motifs, once again with a strong positional preference. Thus, in this case, the preferential arrangement of binding sites on the genome seems to be ZNF143/E-BOX/CCAAT, on either strand, with a precise spacing. Since none of the known E-box binding TFs so far included in the K562 datasets shows relevant cobinding with ZNF143 inside NF-Y peaks, it remains to be determined what could be the actual TF binding the E-Boxes, or if there are different TFs of the same family binding each a subset of them.

**Figure 9:**
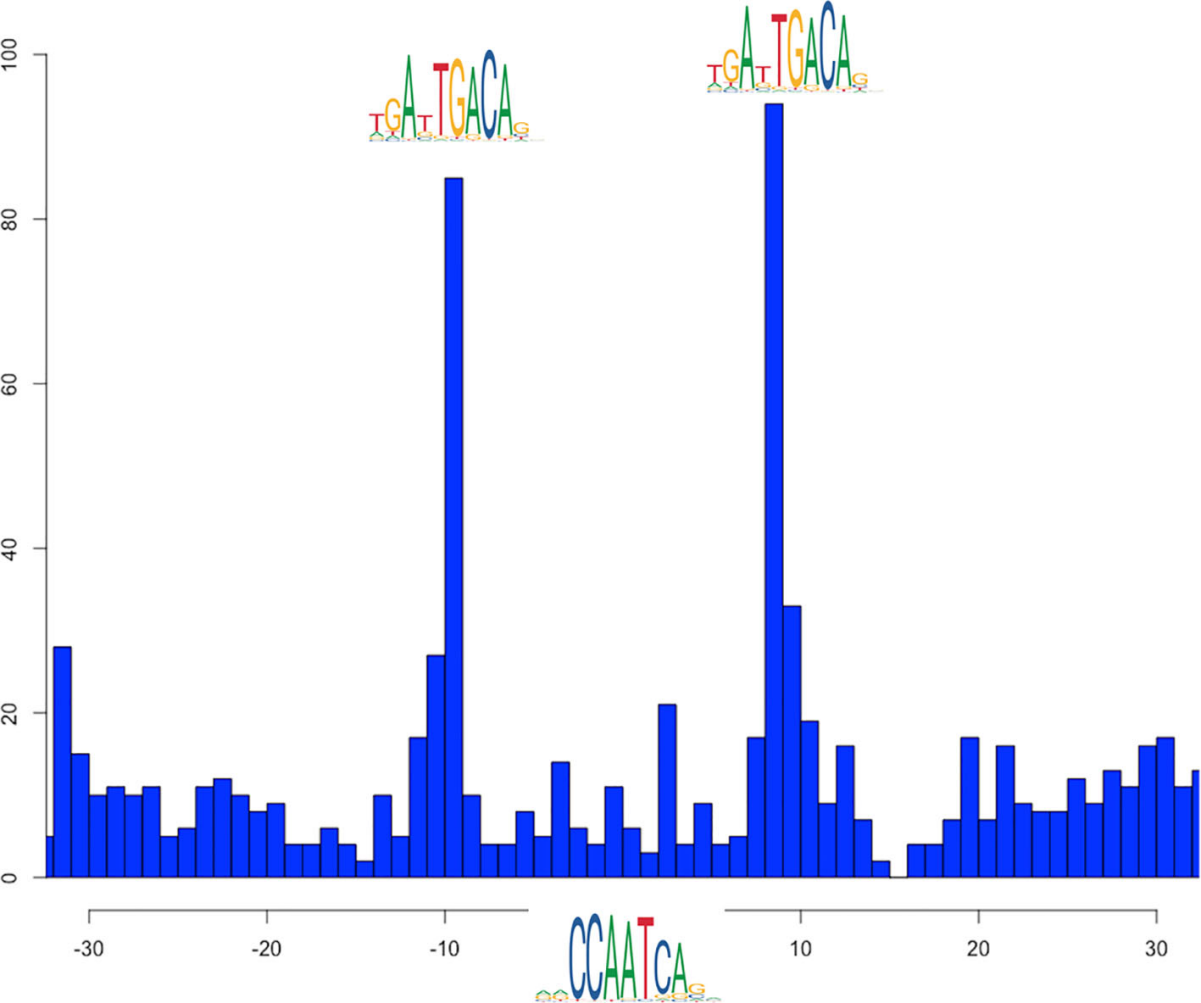
Distribution relative positions of PBX2 binding site with respect to the CCAAT box motif in cobinding peaks of NF-YB with PBX2, as reported by PscanChIP from in the motif centered analysis on the CCAAT box motif (**Supplementary Table 8**). The origin of the x-axis corresponds to the center of the CCAAT box binding sites. The analysis has been performed on 1782 cobinding regions for NF-YB and ZNF143.

A final example is colocalizing peaks with precise motif arrangement of NF-YB with PBX2 (in turn with significant overlap with PKNOX1, green cluster in **Figure 4**): the respective binding sites on the genome can be found, once again with a clear distance preference (**Figure 9**). The interaction of NF-Y with TALE transcription factors, including PBX2, and the arrangement of their binding sites on DNA has indeed been recently reported as for example in zebrafish (Ladam et al., 2018). In this case, however, PscanChIP motif analysis reports that in about 20% of the cobinding peaks the CCAAT box motif is returned to be the most likely candidate also for the binding of PBX2, since its consensus motif (CTGTCAATCA) in turn contains a CAAT subsequence (see **Supplementary Table 8**). Also, the p-value associated by PscanChIP to the PBX2 motif is only marginally significant. Thus, it remains to be ascertained whether the binding motifs found on DNA are actually bound by the respective transcription factors in all the cobinding regions, or, as more likely, there are instances where a single or double CCAAT box bound by NF-Y is the motif tethering the complex on DNA.

## Discussion

We presented a computational pipeline that, starting from a collection of peak regions resulting from the analysis of different TFs and cofactors, is able to single out the most relevant TF combinations and modules in the condition studied. The integration of peak and summit overlap with a sequence analysis method developed specifically for the analysis of ChIP-Seq regions also permits the characterization of the recruitment rules on DNA for the complex and the organization of the respective binding sites on the genome.

A preliminary version of this pipeline has been applied to the analysis of the complete collection of ENCODE ChIP-Seq experiments in three different cell lines, focusing on modules containing transcription factor NF-Y. In this work, we reanalyzed the updated ENCODE data for K562, essentially confirming the previous results for NF-Y, as well as finding novel candidate interactors and genome-wide coassociations involving also FOS, USF1, and USF2. We are now working on manuscripts detailing the results obtained also on additional cell lines, on different TFs and cofactors, as well as linking these findings to functionality.

Our approach permits to build a picture of the regulatory landscape of a given condition, highlighting the TF coassociations found more frequently, and assessing their significance as well as the corresponding organization of binding sites on the genome. It can be integrated with additional sources of information. For example, one could focus on active promoters or enhancers, as revealed by presence of specific histone marks, and restrict the cobinding peak analysis only to those regions that carry a precise chromatin annotation, resulting for example from a genome segmentation approach (Hoffman et al., 2009; Ernst and Kellis, 2017). In this way separate maps of regulatory modules specific for enhancers and/or promoters can be built. The data can be complemented with RNA-Seq experiments performed after inactivation of the single TF, so that the functionality—positive, negative, or neutral—of the single modules can be inferred. Finally, the exact pattern of binding in a single selected region can be further analyzed by employing more sophisticated sequence analysis approaches (Gheorghe et al., 2019).

## Data Availability Statement

Publicly available datasets were analyzed in this study. This data can be found at the Encode Project (www.encodeproject.org) and the accession numbers are listed in **Supplementary Material**.

## Author Contributions

GP and RM devised the original pipeline, that has been fine tuned and modified with input from DD, MR, and FZ. MR and FZ implemented different parts of the pipeline, supervised by GP. MR run the analyses presented in the article and collected the results. All authors examined and discussed the results.

## Funding

Consiglio Nazionale delle Ricerche, Flagship Research Project “EPIGEN.”

## Conflict of Interest

The authors declare that the research was conducted in the absence of any commercial or financial relationships that could be construed as a potential conflict of interest.

## Supplementary Material

Supplementary File 1 Shell script implementing the key steps of the pipeline through the “bedtools” utility (version 2.29).

Supplementary Table 1 List of ENCODE identifiers of the ChIP-Seq datasets employed in this study.

Supplementary Table 2 Results of the peak/summit overlap step between all pairs of ENCODE datasets in the K562 cell line.

Supplementary Table 3 Results of the peak/summit overlap step between all pairs of ENCODE datasets in the K562 cell line within NF-YB peaks.

Supplementary Table 4 Results of the peak/summit overlap step between all pairs of ENCODE datasets in the K562 cell line within FOS peaks.

Supplementary Table 5 Results of the peak/summit overlap step between all pairs of ENCODE datasets in the K562 cell line within USF1 peaks.

Supplementary Table 6 List of co-binding summit coordinates for NF-YB and USF1, complete PscanChIP output, and relative position of NF-YB binding sites with respect to USF1 sites as output by PscanChIP.

Supplementary Table 7 List of co-binding summit coordinates for NF-YB and ZNF143, complete PscanChIP output, and relative position of NF-YB and E-Box binding sites with respect to ZNF143 sites as output by PscanChIP.

Supplementary Table 8 List of co-binding summit coordinates for NF-YB and PBX2, complete PscanChIP output, and relative position of NF-YB with respect to PBX2 sites as output by PscanChIP.

## References

[B1] Aperlo C. Boulukos K. E. Pognonec P. (1996). The basic region/helix-loop-helix/leucine repeat transcription factor USF interferes with Ras transformation. Eur. J. Biochem. 241, 249–253. 10.1111/j.1432-1033.1996.0249t.x 8898913

[B2] Bailey T. L. MacHanick P. (2012). Inferring direct DNA binding from ChIP-seq. Nucleic Acids Res. 40 (17), e128. 10.1093/nar/gks433 22610855 PMC3458523

[B3] Blanar M. A. Rutter W. J. (1992). Interaction cloning: identification of a helix-loop-helix zipper protein that interacts with c-Fos. Science (80-), 1014–1018. 10.1126/science.1589769 1589769

[B4] Buenrostro J. D. Giresi P. G. Zaba L. C. Chang H. Y. Greenleaf W. J. (2013). Transposition of native chromatin for fast and sensitive epigenomic profiling of open chromatin, DNA-binding proteins and nucleosome position. Nat. Methods 10, 1213–1218. 10.1038/nmeth.2688 24097267 PMC3959825

[B5] Celona B. Weiner A. Di Felice F. Mancuso F. M. Cesarini E. Rossi R. L. (2011). Substantial Histone reduction modulates Genomewide nucleosomal occupancy and global transcriptional output. PloS Biol. 9 (6), e1001086. 10.1371/journal.pbio.1001086 21738444 PMC3125158

[B6] Chèneby J. Gheorghe M. Artufel M. Mathelier A. Ballester B. (2018). ReMap 2018: an updated atlas of regulatory regions from an integrative analysis of DNA-binding ChIP-seq experiments. Nucleic Acids Res. 46, D267–D275. 10.1093/nar/gkx1092 29126285 PMC5753247

[B7] Chen X. Xu H. Yuan P. Fang F. Huss M. Vega V. B. (2008). Integration of external signaling pathways with the core transcriptional network in embryonic stem cells. Cell 133, 1106–1117. 10.1016/j.cell.2008.04.043 18555785

[B8] Czipa E. Schiller M. Nagy T. Kontra L. Steiner L. Koller J. (2020). ChIPSummitDB: a ChIP-seq-based database of human transcription factor binding sites and the topological arrangements of the proteins bound to them. Database 2020, baz141. 10.1093/database/baz141 31942977 PMC6964213

[B9] Davis C. A. Hitz B. C. Sloan C. A. Chan E. T. Davidson J. M. Gabdank I. (2018). The encyclopedia of DNA elements (ENCODE): data portal update. Nucleic Acids Res. 46, D794–D801. 10.1093/nar/gkx1081 29126249 PMC5753278

[B10] Dergilev A. I. Spitsina A. M. Chadaeva I. V. Svichkarev A. V. Naumenko F. M. Kulakova E. V. (2017). Computer analysis of colocalization of the TFs’ binding sites in the genome according to the ChIP-seq data. Russ. J. Genet. Appl. Res. 7, 513–522. 10.1134/S2079059717050057

[B11] Dolfini D. Zambelli F. Pedrazzoli M. Mantovani R. Pavesi G. (2016). A high definition look at the NF-Y regulome reveals genome-wide associations with selected transcription factors. Nucleic Acids Res. 44, 4684–4702. 10.1093/nar/gkw096 26896797 PMC4889920

[B12] Ernst J. Kellis M. (2017). Chromatin-state discovery and genome annotation with ChromHMM. Nat. Protoc. 12, 2478–2492. 10.1038/nprot.2017.124 29120462 PMC5945550

[B13] Fleming J. D. Pavesi G. Benatti P. Imbriano C. Mantovani R. Struhl K. (2013). NF-Y coassociates with FOS at promoters, enhancers, repetitive elements, and inactive chromatin regions, and is stereo-positioned with growth-controlling transcription factors. Genome Res. 23, 1195–1209. 10.1101/gr.148080.112 23595228 PMC3730095

[B14] Fullwood M. J. Ruan Y. (2009). ChIP-based methods for the identification of long-range chromatin interactions. J. Cell Biochem. 107, 30–39. 10.1002/jcb.22116 19247990 PMC2748757

[B15] Gerstein M. B. Kundaje A. Hariharan M. Landt S. G. Yan K. K. Cheng C. (2012). Architecture of the human regulatory network derived from ENCODE data. Nature 489, 91–100. 10.1038/nature11245 22955619 PMC4154057

[B16] Gheorghe M. Sandve G. K. Khan A. Chèneby J. Ballester B. Mathelier A. (2019). A map of direct TF-DNA interactions in the human genome. Nucleic Acids Res. 47, e21. 10.1093/nar/gky1210 30517703 PMC6393237

[B17] Giresi P. G. Kim J. McDaniell R. M. Iyer V. R. Lieb J. D. (2007). FAIRE (formaldehyde-assisted isolation of regulatory elements) isolates active regulatory elements from human chromatin. Genome Res. 17, 877–885. 10.1101/gr.5533506 17179217 PMC1891346

[B18] Harwood J. C. Kent N. A. Allen N. D. Harwood A. J. (2019). Nucleosome dynamics of human iPSC during neural differentiation. EMBO Rep. 20 (6), e46960. 10.15252/embr.201846960 31036712 PMC6549019

[B19] Haubrock M. Hartmann F. Wingender E. (2016). NF-Y binding site architecture defines a C-Fos targeted promoter class. PloS One 11 (8), e0160803. 10.1371/journal.pone.0160803 27517874 PMC4982600

[B20] Heinz S. Benner C. Spann N. Bertolino E. Lin Y. C. Laslo P. (2010). Simple combinations of lineage-determining transcription factors prime cis-regulatory elements required for macrophage and B cell identities. Mol. Cell 38, 576–589. 10.1016/j.molcel.2010.05.004 20513432 PMC2898526

[B21] Hoffman M. M. Buske O. Bilmes J. Noble W. (2009). Segway: simultaneous segmentation of multiple functional genomics data sets with heterogeneous patterns of missing data. NobleGsWashingtonEdu 2–5.

[B22] Johnson D. S. Mortazavi A. Myers R. M. Wold B. (2007). Genome-wide mapping of in vivo protein-DNA interactions. Science (80-), 1497–1502. 10.1126/science.1141319 17540862

[B23] Kanduri C. Bock C. Gundersen S. Hovig E. Sandve G. K. (2019). Colocalization analyses of genomic elements: approaches, recommendations and challenges. Bioinformatics 35, 1615–1624. 10.1093/bioinformatics/bty835 30307532 PMC6499241

[B24] Khan A. Fornes O. Stigliani A. Gheorghe M. Castro-Mondragon J. A. Van Der Lee R. (2018). JASPAR 2018: update of the open-access database of transcription factor binding profiles and its web framework. Nucleic Acids Res. 46, D260–D266. 10.1093/nar/gkx1126 29140473 PMC5753243

[B25] Ladam F. Stanney W. Donaldson I. J. Yildiz O. Bobola N. Sagerström C. G. (2018). TALE factors use two distinct functional modes to control an essential zebrafish gene expression program. Elife 7, e36144. 10.7554/eLife.36144 29911973 PMC6023610

[B26] Landt S. G. Marinov G. K. Kundaje A. Kheradpour P. Pauli F. Batzoglou S. (2012). ChIP-seq guidelines and practices of the ENCODE and modENCODE consortia. Genome Res. 22, 1813–1831. 10.1101/gr.136184.111 22955991 PMC3431496

[B27] Levitsky V. Zemlyanskaya E. Oshchepkov D. Podkolodnaya O. Ignatieva E. Grosse I. (2019). A single ChIP-seq dataset is sufficient for comprehensive analysis of motifs co-occurrence with MCOT package. Nucleic Acids Res. 47(21), e139. 10.1093/nar/gkz800 31750523 PMC6868382

[B28] Lieberman-Aiden E. Van Berkum N. L. Williams L. Imakaev M. Ragoczy T. Telling A. (2009). Comprehensive mapping of long-range interactions reveals folding principles of the human genome. Science (80-), 289–293. 10.1126/science.1181369 PMC2858594 19815776

[B29] Machanick P. Bailey T. L. (2011). MEME-ChIP: Motif analysis of large DNA datasets. Bioinformatics 27, 1696–1697. 10.1093/bioinformatics/btr189 21486936 PMC3106185

[B30] Mathelier A. Fornes O. Arenillas D. J. Chen C. Y. Denay G. Lee J. (2016). JASPAR 2016: A major expansion and update of the open-access database of transcription factor binding profiles. Nucleic Acids Res. 44, D110–D115. 10.1093/nar/gkv1176 26531826 PMC4702842

[B31] Oki S. Ohta T. Shioi G. Hatanaka H. Ogasawara O. Okuda Y. (2018). ChIP -Atlas: a data-mining suite powered by full integration of public ChIP -seq data. EMBO Rep. 19 (12), e46255. 10.15252/embr.201846255 30413482 PMC6280645

[B32] Pajoro A. Muiño J. M. Angenent G. C. Kaufmann K. (2018). “Profiling nucleosome occupancy by MNase-seq: experimental protocol and computational analysis,” in Methods in molecular biology, (New York, NY:Humana Press), 167–181. 10.1007/978-1-4939-7318-7_11 29052192

[B33] Quinlan A. R. Hall I. M. (2010). BEDTools: a flexible suite of utilities for comparing genomic features. Bioinformatics 26, 841–842. 10.1093/bioinformatics/btq033 20110278 PMC2832824

[B34] Roadmap Epigenomics Consortium Kundaje A. Meuleman W. Ernst J. Bilenky M. Yen A. (2015). Integrative analysis of 111 reference human epigenomes. Nature 518, 317–330. 10.1038/nature14248 25693563 PMC4530010

[B35] Sabo P. J. Hawrylycz M. Wallace J. C. Humbert R. Yu M. Shafer A. (2004). Discovery of functional noncoding elements by digital analysis of chromatin structure. Proc. Natl. Acad. Sci. U. S. A. 101, 16837–16842. 10.1073/pnas.0407387101 15550541 PMC534745

[B36] Salvatore S. Dagestad Rand K. Grytten I. Ferkingstad E. Domanska D. Holden L. (2019). Beware the Jaccard: the choice of similarity measure is important and non-trivial in genomic colocalisation analysis. Brief Bioinform. 10.1093/bib/bbz083 31624847

[B37] Stormo G. D. (2000). DNA binding sites: representation and discovery. Bioinformatics 16, 16–23. 10.1093/bioinformatics/16.1.16 10812473

[B38] Tannenbaum M. Sarusi-Portuguez A. Krispil R. Schwartz M. Loza O. Benichou J. I. C. (2018). Regulatory chromatin landscape in Arabidopsis thaliana roots uncovered by coupling INTACT and ATAC-seq. Plant Methods 14, 113. 10.1186/s13007-018-0381-9 30598689 PMC6300899

[B39] Thomas S. Li X. Y. Sabo P. J. Sandstrom R. Thurman R. E. Canfield T. K. (2011). Dynamic reprogramming of chromatin accessibility during Drosophila embryo development. Genome Biol. 12. 10.1186/gb-2011-12-5-r43 PMC3219966 21569360

[B40] Thomas R. Thomas S. Holloway A. K. Pollard K. S. (2017). Features that define the best ChIP-seq peak calling algorithms. Brief Bioinform. 18, 441–450. 10.1093/bib/bbw035 27169896 PMC5429005

[B41] Vierstra J. Stamatoyannopoulos J. A. (2016). Genomic footprinting. Nat. Methods 13, 213–221. 10.1038/nmeth.3768 26914205

[B42] Wang J. Zhuang J. Iyer S. Lin X. Y. Whitfield T. W. Greven M. C. (2012). Sequence features and chromatin structure around the genomic regions bound by 119 human transcription factors. Genome Res. 22, 1798–1812. 10.1101/gr.139105.112 22955990 PMC3431495

[B43] Wingender E. Schoeps T. Haubrock M. Krull M. Dönitz J (2018). TFClass: expanding the classification of human transcription factors to their mammalian orthologs. Nucleic Acids Res. 46, D343–D347. 10.1093/nar/gkx987 29087517 PMC5753292

[B44] Wingender E. (2008). The TRANSFAC project as an example of framework technology that supports the analysis of genomic regulation. Brief Bioinform. 9, 326–332. 10.1093/bib/bbn016 18436575

[B45] Xie D. Boyle A. P. Wu L. Zhai J. Kawli T. Snyder M. (2013). Dynamic trans-acting factor colocalization in human cells. Cell 155, 713. 10.1016/j.cell.2013.09.043 24243024 PMC4079469

[B46] Zambelli F. Pavesi G. (2017). Genome wide features, distribution and correlations of NF-Y binding sites. Biochim. Biophys. Acta 1860, 581–589. 10.1016/j.bbagrm.2016.10.007 27769808

[B47] Zambelli F. Pesole G. Pavesi G. (2013a). Motif discovery and transcription factor binding sites before and after the next-generation sequencing era. Brief Bioinform. 14, 225–237. 10.1093/bib/bbs016 22517426 PMC3603212

[B48] Zambelli F. Pesole G. Pavesi G. (2013b). PscanChIP: finding over-represented transcription factor-binding site motifs and their correlations in sequences from ChIP-Seq experiments. Nucleic Acids Res. 41, W535–W543. 10.1093/nar/gkt448 23748563 PMC3692095

[B49] Zambelli F. Pesole G. Pavesi G. (2014). Using weeder, Pscan, and PscanChIP for the discovery of enriched transcription factor binding site motifs in nucleotide sequences. Curr. Protoc. Bioinform. 47, 2.11.1–2.1131. 10.1002/0471250953.bi0211s47 25199791

[B50] Zhang Y. Liu T. Meyer C. A. Eeckhoute J. Johnson D. S. Bernstein B. E.17. (2008). Model-based analysis of ChIP-Seq (MACS). Genome Biol. 9, R137. 10.1186/gb-2008-9-9-r137 18798982 PMC2592715

[B51] Zhang Z. Chang C. W. Goh W. L. Sung W. K. Cheung E. (2011). CENTDIST: discovery of co-associated factors by motif distribution. Nucleic Acids Res. 39, W391–W399. 10.1093/nar/gkr387 21602269 PMC3125780

[B52] Zhu J. Giannola D. M. Zhang Y. Rivera A. J. Emerson S. G. (2003). NF-Y cooperates with USF1/2 to induce the hematopoietic expression of HOXB4. Blood 102, 2420–2427. 10.1182/blood-2003-01-0251 12791656

