# Supplementary material for: Integrating Peak Colocalization and Motif Enrichment Analysis for the Discovery of Genome-Wide Regulatory Modules and Transcription Factor Recruitment Rules
Source: Front Genet. 2020 Feb 21;11:72. doi: 10.3389/fgene.2020.00072 (PMC7046753; doi:10.3389/fgene.2020.00072)
Supplement: Supplementary File 1 — Shell script implementing the key steps of the pipeline through the “bedtools” utility (version 2.29). [file DataSheet_1.pdf]

```
#!/bin/sh

# shell script for computing summit overlap between two ChIP-Seq experiments
# input: bed files of ChIP-Seq summits for two TFs ($1 and $2, provided as
arguments to the script). Advised to employ 1-bp summits
# sort the input summit files for TF1 and TF2

sort -k1,1 -k2,2n $1 > $1.sorted.bed
sort -k1,1 -k2,2n $2 > $2.sorted.bed

# return for each peak summit of TF1 its coordinates, the coordinates of the
closest summit of TF2, and its distance (as in bedtools version 2.29).

bedtools closest -a $1.sorted.bed  -b $2.sorted.bed -d > $1_$2_overlap.bed

# select from the overlap file output by bedtools only those summits within 150
bps from each other

awk -F "\t" '$7<=150' $1_$2_overlap.bed > $1_$2_overlap_150.bed

# count the number of co-binding peaks

wc -l $1_$2_overlap_150.bed
```
